# miR-21 upregulation exacerbates pressure overload-induced cardiac hypertrophy in aged hearts

**DOI:** 10.18632/aging.204194

**Published:** 2022-07-28

**Authors:** Wei-Ting Chang, Jhih-Yuan Shih, Yu-Wen Lin, Tzu-Ling Huang, Zhih-Cherng Chen, Chi-Long Chen, Jan-Show Chu, Ping Yen Liu

**Affiliations:** 1 Institute of Clinical Medicine, College of Medicine, National Cheng Kung University, Tainan, Taiwan; 2 Department of Internal Medicine, Division of Cardiology, Chi-Mei Medical Center, Tainan, Taiwan; 3 Department of Biotechnology, Southern Taiwan University of Science and Technology, Tainan, Taiwan; 4 Department of Health and Nutrition, Chia Nan University of Pharmacy and Science, Tainan, Taiwan; 5 Department of Pathology, College of Medicine, School of Medicine, Taipei Medical University, Taipei, Taiwan; 6 Department of Pathology, Taipei Medical University Hospital, Taipei, Taiwan; 7 Department of Internal Medicine, Division of Cardiology, National Cheng Kung University Hospital, College of Medicine, National Cheng Kung University, Tainan, Taiwan

**Keywords:** miR-21, aging, cardiac hypertrophy, hypertension, pressure overload

## Abstract

Young and aging hearts undergo different remodeling post pressure overload, but the regulator that determines responses to pressure overload at different ages remains unknown. With an angiotensin II (Ang II)-induced hypertensive model, miR-21 knockout mice (miR-21^−/−^) were observed regarding the effects of miR-21 on hypertension-induced cardiac remodeling in young (12 week-old) and old (50 week-old) mice. Although the aged heart represented a more significant hypertrophy and was associated with a higher expression of miR-21, Ang II-induced cardiac hypertrophy was attenuated in miR-21^−/−^ mice. Upon results of cardiac-specific arrays in miR-21-overexpressing cardiomyocytes, we found a significant downregulation of S100a8. In both *in vitro* and *in vivo* models, miR-21/S100a8/NF-κB/NFAT pathway was observed to be associated with pressure overload-induced hypertrophic remodeling in aged hearts. To further investigate whether circulating miR-21 could be a biomarker reflecting the aged associated cardiac remodeling, we prospectively collected clinical and echocardiographic information of patients at young (<65 y/o) and old ages (≥65 y/o) with and without hypertension. Among 108 patients, aged subjects presented with a significantly higher expression of circulating miR-21, which was positively correlated with left ventricular wall thickness. Collectively, miR-21 was associated with a prominently hypertrophic response in aged hearts under pressure overload. Further studies should focus on therapeutic potentials of miR-21.

## INTRODUCTION

Aging is a major cause of congestive heart failure [1, 2]. More than 75% of patients with congestive heart failure are over age 65, which in the elderly contributes to a significant increase in cardiovascular mortality and heart failure [3]. However, cardiac aging with concurrent hypertension often causes cardiomyopathy, which has not been well studied. Physiologically, diastolic heart failure appears when the ventricle cannot be filled properly, since its wall is too rigid and fails to relax properly [4, 5]. Histological evidence has indicated continuous loss of myocytes, compensation by reactive hypertrophy of the remaining cells and filling with interstitial fibrosis [1, 5]. In our previous studies, we found that, in young and aging populations, cardiac remodeling under pressure overload varied [6], while a single transduction pathway may hardly explain the extensively involved hypertrophic, fibrotic and apoptotic cascades. However, microRNAs (miRNAs), noncoding RNA species that regulate posttranscriptional modification, may consequently regulate the activation of various pathways [7]. Among them, miR-21 has been shown to play an important role in myocardial fibrosis and heart failure [8]. A previous study found that overexpression of miR-21 markedly blocked Ang II-induced cardiac hypertrophy by targeting histone deacetylase-8 [9]. In contrast, Patrick et al. found that inhibition of miR-21 in mice contributed to less interstitial fibrosis and improvement of cardiac function in a pressure overload cardiac disease mouse model [10]. Therefore, the definite role of miR-21 remains controversial. Herein, through both clinical observation and a hypertensive mouse model, we aimed to elucidate the regulatory role of miR-21 in the remodeling of young and aged hearts facing hypertension-induced pressure overload.

## MATERIALS AND METHODS

### Mouse model of hypertension

All animal experiments followed guidelines of Care and Use of Laboratory Animals. All animal protocols were approved by Subcommittee on Research Animal Care of Chi-Mei Medical Center. miR-21 knockout (miR-21^−/−^) mice in C57BL/6J background and wild type were purchased from Jackson Laboratory (Charles River, Boston, MA, USA). Mice were maintained on a 12 hr light/dark cycle and they had free access to water and food in Animal Resource Center of Chi-Mei Medical Center. 12-week-old (young) and 50-week-old (aged) male miR-21^−/−^ and wild type mice were used in the present study and randomly assigned to (1) control young group, (2) control aged group, (3) Ang II young group, and (4) Ang II aged group. Previous literature also defined 50-week old mice as the aged mice [11, 12]. For the hypertensive model, Alzet osmotic micropumps (model 2004, Durect Corporation, Cupertino, CA, USA) were subcutaneously implanted into mice [13]. Each pump delivered 1000 ng/kg/min of Ang II (Millipore-Sigma, USA) at a rate of 0.25 μL/h during 28 days. The rats’ survival rate, cardiac function, and blood pressure were measured weekly. The detailed experimental design is shown in Supplementary Figure 1. The details of histopathological characterization, mouse echocardiography and administration of miR-21 antagomir were displayed in Supplementary Materials and Methods.

### Primary adult mouse cardiomyocyte isolation

After euthanasia, the mouse hearts were cannulated to the Langendorff Apparatus through the aorta to the coronary arteries. As previously described [14], the tissue was perfused with calcium-free Krebs buffer at a steady temperature of 37°C followed by buffer containing Pronase and Collagenase (Sigma-Aldrich, St. Louis, MO, USA). Subsequently, using dissecting forceps the digested tissues were separated into small pieces. After filtering the digested tissue through a squared mesh, the live cells were collected at the bottom of the tube. After going through the calcium gradient buffer, cardiomyocytes were successfully isolated.

### Neonatal cardiomyocyte isolation and cell culture

Neonatal rat cardiomyocytes were isolated from newborn rats by enzymatic digestion as described previously [15]. Briefly, neonatal cardiomyocytes were cultivated in minimal essential medium (Animed) containing vitamin B12, NaHCO_3_, L-glutamine, penicillin/streptomycin, and 5% FBS (Invitrogen, Thermo Fisher Scientific, Waltham, MA, USA) at 37°C in 1% CO_2_. Culture medium was changed every 2 days. The cultured cardiomyocytes were allowed to grow for 2 day as young cardiomyocytes or 14 days as aged cardiomyocytes. The details of β-galactosidase staining, transfection of miR-21 mimic and inhibitor and measurement of hypertrophy in cardiomyocytes were listed in Supplementary Materials and Methods.

### RNA isolation and quantitative real time-polymerase chain reaction

Total RNA was isolated from myocardium, primarily isolated adult and neonatal cardiomyocytes with Trizol (Ambion). cDNA was generated using the Taqman MicroRNA Assays (Foster City, CA). Primer sequences used in the present study were shown in Supplementary Table 1. The details of β-galactosidase staining, transfection of miR-21 mimic and inhibitor and measurement of quantitative PCR (qPCR) array was listed in Supplementary Materials and Methods.

### Patients and study design

We prospectively collected the clinical and echocardiographic information of 108 patients in various ages (≧65 y/o defined as the aged group) with or without hypertension. The exclusion criteria consist of (1) impaired LV systolic function at baseline (left ventricular ejection fraction less than 40%) [16, 17], (2) age <8 or >80 year-old and (3) medical records of diabetes, coronary arterial disease, symptomatic heart failure, hypertrophic cardiomyopathy, significant (above moderate severity) valvular heart disease or other major organ dysfunction. All hypertensive patients were newly diagnosed and treatment naïve and the echocardiography was performed at diagnosis. The study was conducted in strict accordance with the Declaration of Helsinki on Biomedical Research involving human subjects and was approved by the local ethics committee (IRB: 10307–003). Human blood sampling and echocardiography were displayed in Supplementary Materials and Methods.

### Statistical analysis

Continuous data are presented as the mean ± standard deviation (S.D.). Dichotomous data are presented as numbers and percentages. Group differences were analyzed using analysis of variance (ANOVA). Significant differences between groups were verified using a Tukey post hoc test. Chi-square tests or Fisher’s exact tests were used for the categorical variables as appropriate. Linear correlation including R was analyzed to represent the correlation between two continuous parameters. Significance was set at *p* < 0.05. The Statistical Package for the Social Sciences (SPSS) software (version 22.0, IBM SPSS Inc., Chicago, IL, USA) and GraphPad Prism (Version 5.03) were used for the statistical analyses.

### Availability of data and materials

The data is available upon the reasonable request to the corresponding author.

## RESULTS

### Cardiac expression of miR-21 is associated with aging-specific cardiac hypertrophy post Ang II treatment

**Figure 1:**
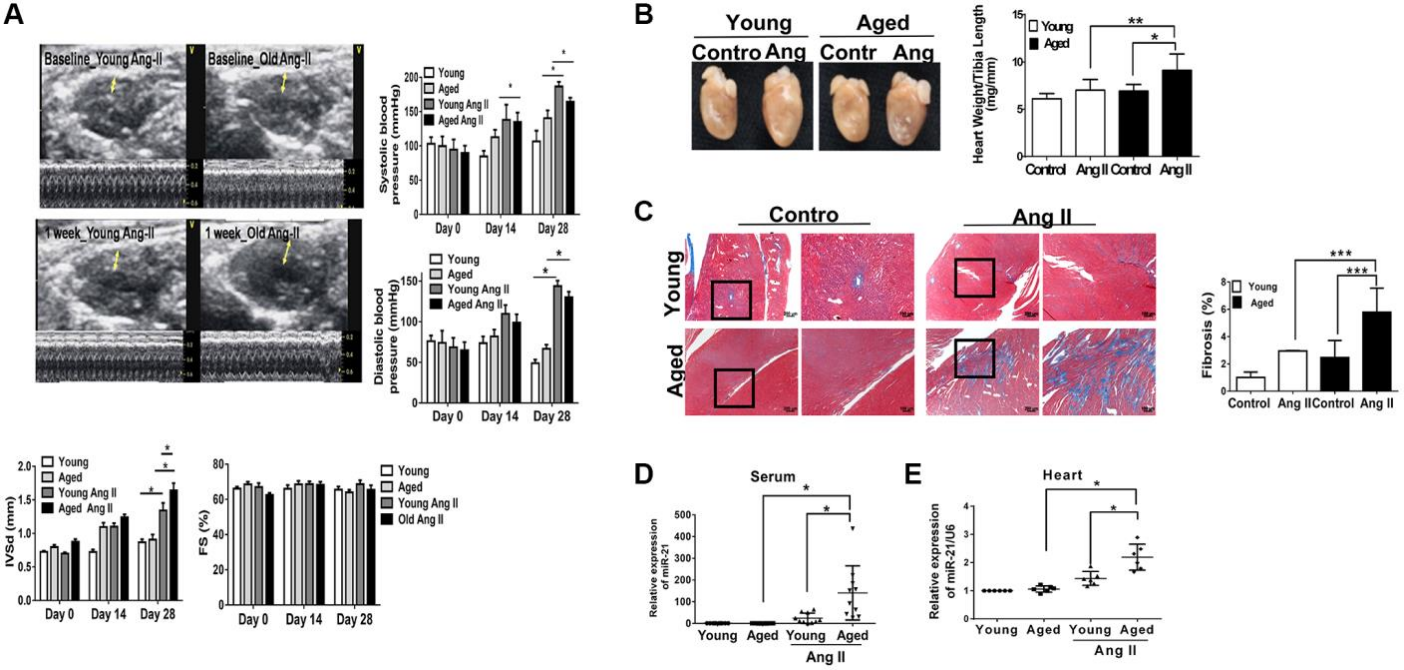
**Angiotensin II (Ang II)-induced cardiac hypertrophy and fibrosis, especially in aged mice.** (**A**) Echocardiography measurements are shown in young and aged mice with or without Ang II. Systolic and diastolic blood pressures recorded. Echocardiographic measurements of intraventricular septal thickness at diastole (IVSd) and ejection fraction and fractional shortening. (**B**) Quantitative analysis of heart weight/tibia length. (**C**) Representative sections and amplified images of the highlighted area of hearts stained with Masson's trichrome for fibrosis detection (blue); scale bars, 30 μm (left panel). Quantification of cardiac fibrosis in the indicated groups of rats (right panel). Expression of (**D**) circulating and (**E**) heart tissue expression of miR-21 in mice. Data are expressed using mean ± standard deviation (S.D.). ^*^P < 0.05, ^**^P < 0.01, ^***^P < 0.001 for difference from each group (*N* = 6–12).

Using Ang II micropumps, we established a pressure overload mouse model in young (12 week-old) and aged (50 week-old) mice (Figure 1A). There were no significant changes in body weight, heart rate, or left ventricular internal diameter at diastole (LVIDd) in young and aged mice with or without Ang II micropumps (Supplementary Figure 2). Under Ang II infusion, both systolic and diastolic blood pressures increased in mice at different ages (Figure 1A). Although the increases in blood pressure were significant in the younger mice compared with those in the aged mice, after Ang II treatment for 28 days, cardiac hypertrophy developed most significantly in the aged mouse heart, while the ejection fraction (EF) and fractional shortening (FS) were not significantly changed. Likewise, at the end of the experiment, the ratio of heart weight/tibia length indicated a significant elevation post Ang II treatment, especially in the aged mice (Figure 1B). Masson’s trichrome staining for cardiac fibrosis revealed a significant increase in cardiac fibrosis in mice post Ang II treatment, especially in aged mice, compared with those in the control groups (Figure 1C). Further, there were significantly increasing miR-21 expression in mouse serum (Figure 1D) and heart tissues (Figure 1E) in the Ang II treatment groups. The level of miR-21 in aged mice was higher than that in young mice. Taken together, these results indicated that aged hearts were more prone to cardiac hypertrophy and fibrosis under pressure overload stimulation. Also, miR-21 expression may correlate with the augmenting response toward pressure overload and result in the upregulation of fibrosis and hypertrophy.

### The regulatory role of miR-21 in the response to hypertension in aged hearts

**Figure 2:**
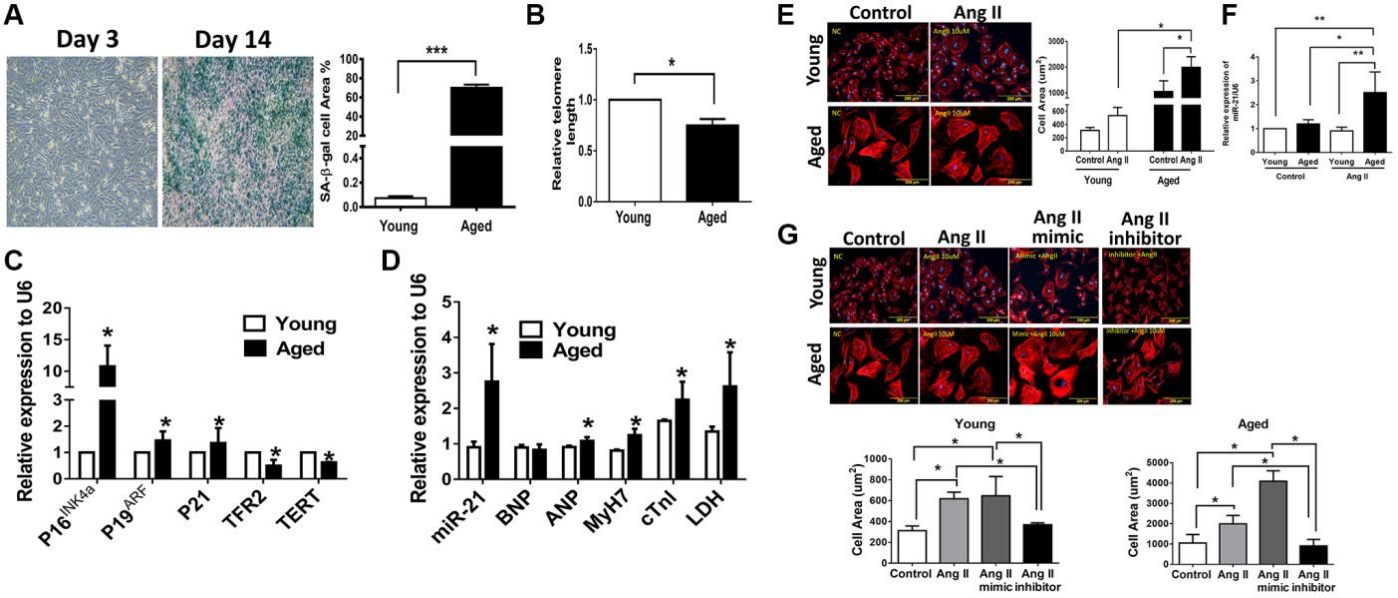
**Angiotensin II (Ang II)-induced more cardiac hypertrophy and miR-21 expression in primarily isolated aged cardiomyocytes than in primary young cardiomyocytes.** (**A**) The cultured neonatal rat cardiomyocytes for 14 days displayed cardiomyocytes senescence. SA-*β*-gal staining results for cardiomyocytes of neonatal rats. Blue precipitation in the cytoplasm was observed in the senescent cells. Percentage of *β*-gal-positive cardiomyocytes was increased in cultured cardiomyocytes for 14 days. (**B**) Telomere length expression in cardiomyocytes of neonatal rats. (**C**) The expression of cell senescence-associated protein in cultured neonatal rat cardiomyocytes was detected by qRT-PCR. (**D**) The levels of miR-21 and cardiac injury-associated genes in cultured neonatal rat cardiomyocytes were detected by qRT-PCR. (**E**) Immunofluorescence assay of F-actin was performed to identify the cell area in each group. Bar charts showing the individual cardiomyocyte cell areas. (**F**) The levels of miR-21 in cultured young and aged rat cardiomyocytes with and without treatment of Ang II detected by qRT-PCR. (**G**) Primarily isolated young and aged cardiomyocytes were transfected with a miR-21 mimic or inhibitor for 24 hours. Representative merged images of F-actin immunofluorescence staining of cardiomyocytes. Overexpression of miR-21 enhanced Ang II-induced cardiac hypertrophy, especially in primarily isolated aged cardiomyocytes. ^*^P < 0.05, ^**^P < 0.01, and ^***^P < 0.001 for difference from each group (*N* = 6–8).

To elucidate the regulatory mechanism of miR-21 in age-differentiated cardiac remodeling under hypertensive conditions, mouse cardiomyocytes from adult mice at different ages (young [12 w/o] and aged [50 w/o]) were primarily isolated (Supplementary Figure 3A). After Ang II treatment, there were significant increases in cell area, especially in cardiomyocytes isolated from aged mice (Supplementary Figure 3B). Notably, miR-21 expression was significantly higher in cardiomyocytes isolated from aged mice than in cardiomyocytes isolated from young mice post Ang II treatment (Supplementary Figure 3C). Although we initially attempted to overexpress miR-21 in primarily isolated cardiomyocytes, the cardiomyocytes isolated from adult mice were too fragile to tolerate transfection with miR-21 mimics. Alternatively, we used neonatal cardiomyocytes isolated from newborn rats (2 days postnatal) instead. Cardiomyocytes cultured for 14 days were defined as aged cardiomyocytes and displayed an increasing number of *β*-gal-positive cells; increased P16^INK4a^, P19^ARF^, and P21; and reduced telomeric repeat-binding factor 2 (TRF2) and telomerase reverse transcriptase (TERT) expression, which implied an aging phenomenon in these cells (Figure 2A–2C). In addition, the levels of miR-21 and cardiac-associated proteins, including atrial natriuretic peptide (ANP), myosin heavy chain 7 (MyH7), cardiac-specific Troponin I (cTnl), and lactic dehydrogenase (LDH), were significantly higher in aged cardiomyocytes than in young cardiomyocytes (Figure 2D).

### Overexpression of miR-21 promotes Ang II-induced hypertrophy, especially in aged cardiomyocytes

Upon our hypothesis that miR-21 is a major regulator controlling age-associated cardiac hypertrophy, we treated aged and young cardiomyocytes with Ang II and found that, post treatment, both primarily isolated young and aged cardiomyocytes presented with hypertrophic changes. Notably, the cell area was most significantly increased in the aged cardiomyocytes (Figure 2E) along with a significant elevation of miR-21 expression (Figure 2F). Using the miR-21 mimic, we found that augmentation of Ang II-induced hypertrophic changes in cardiomyocytes (Figure 2G). Conversely, the suppression of miR-21 reversed Ang II-induced hypertrophy. This phenomenon was observed in both young and aged cardiomyocytes. The levels of BNP and ANP were used to assess the extent of myocardial hypertrophy. The miR-21 mimic induced the expression levels of miR-21, BNP, ANP, and cTnI, which were decreased by the miR-21 inhibitor (Supplementary Figure 4).

### Abolishing miR-21 attenuated Ang II-induced cardiac hypertrophy and fibrosis in both young and aged mice

**Figure 3:**
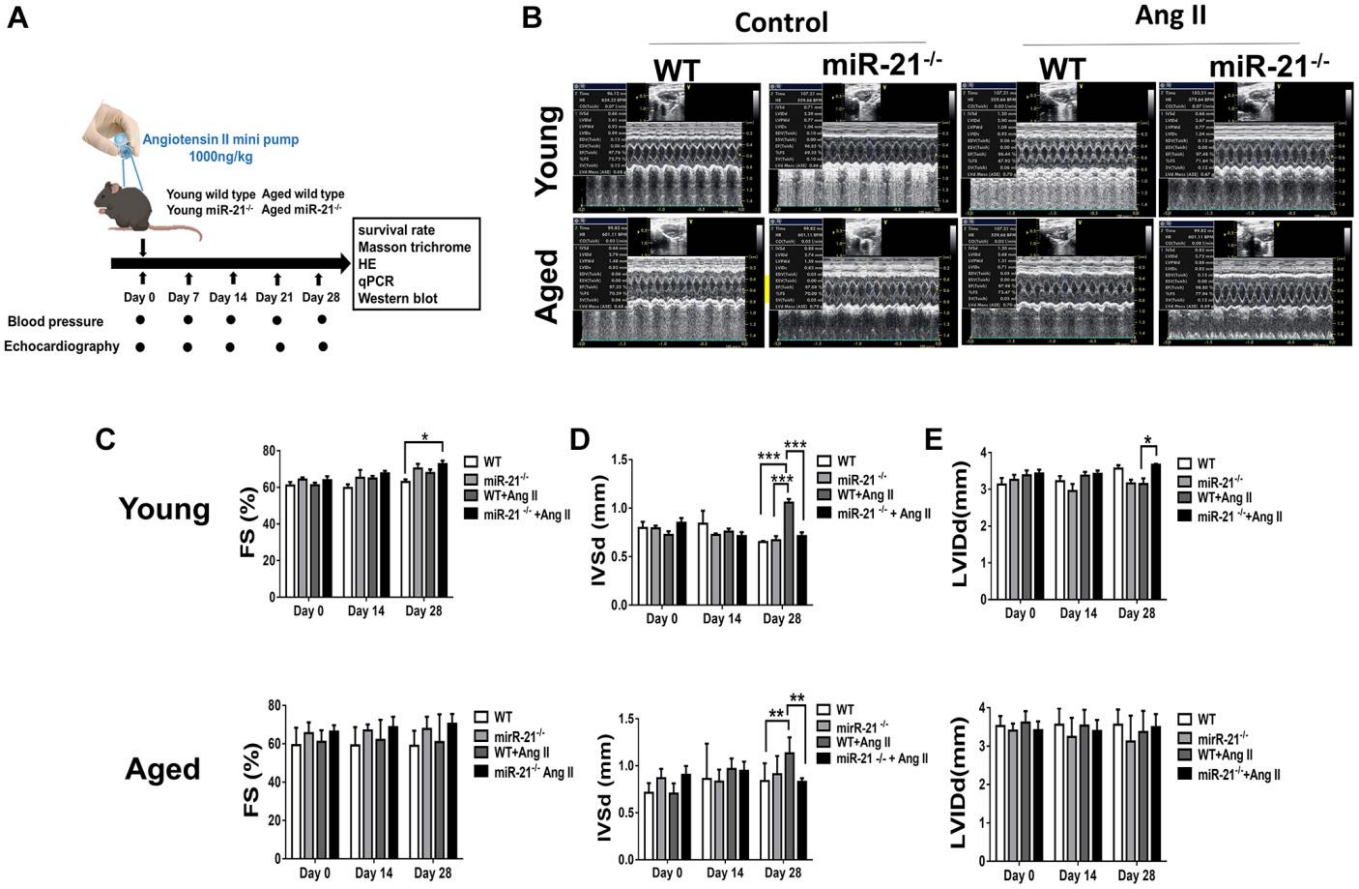
**miR-21 knockout (miR-21^−/−^) protects against angiotensin II (Ang II)-induced cardiac alterations in both young and aged mice.** (**A**) The study design. (**B**) Sequential echocardiography measurements are shown in wild-type (WT) and miR-21^**−/−**^ mice with or without exposure to Ang II. Echocardiographic measurements of (**C**) fractional shortening (FS), (**D**) interventricular septum (IVSd), and (**E**) left ventricular internal diameter in diastole (LVIDd) are shown for each group. ^*^P < 0.05, ^**^P < 0.01, and ^***^P < 0.001 for difference from each group (*N* = 6–8).

**Figure 4:**
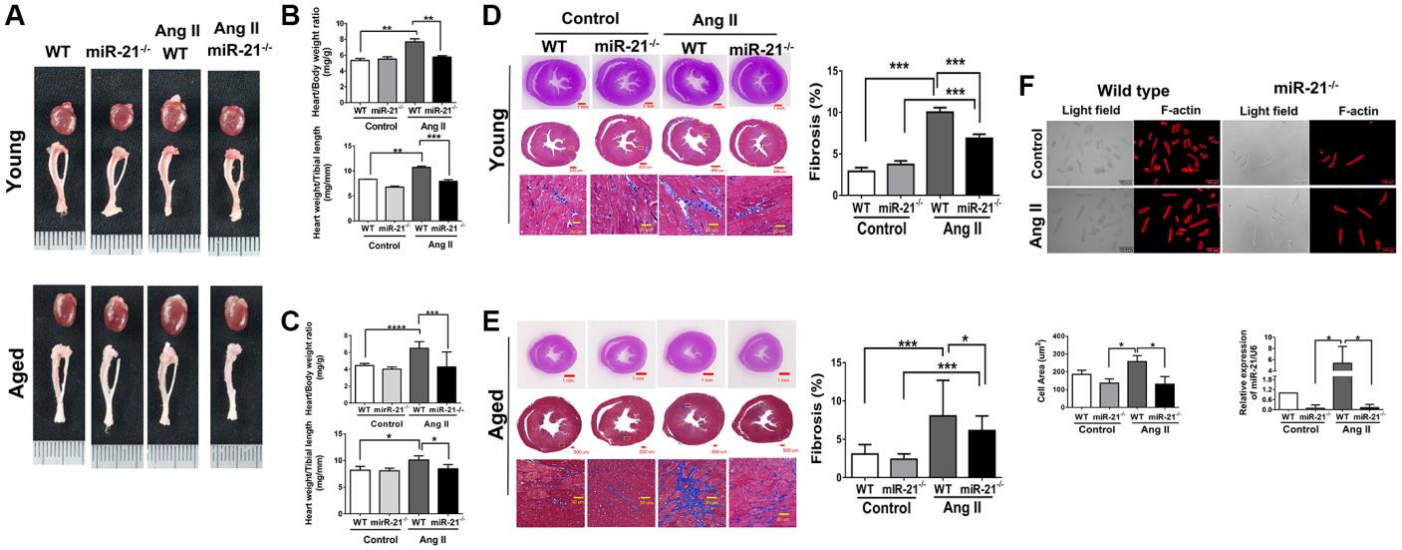
**miR-21 knockout (miR-21^−/−^) decreased angiotensin II (Ang II)-induced cardiac hypertrophy and fibrosis in both young and aged mice.** (**A**) Representative images of harvested hearts. Quantitative analysis of heart weight/body weight and heart weight/tibia length in wild type (WT) or miR-21^−/−^ of (**B**) young and (**C**) aged mice. In WT or miR-21^−/−^ of (**D**) young and (**E**) aged mice, representative sections of hearts stained with Masson's trichrome for fibrosis detection (blue); scale bars, 30 μm (left panel). Quantification of cardiac fibrosis (right panel). (**F**) miR-21^−/−^ decreased Ang II-induced increased cardiac hypertrophy miR-21 expression in primary mouse cardiomyocyte. The representative merged images of light field and F-actin immunofluorescence staining for primary cardiomyocyte isolated from WT and miR-21^−/−^ of young mice. The cell area was measured 100 random cells in each group. The expression of miR-21 was measured by qRT-PCR in each group. ^*^P < 0.05, ^**^P < 0.01, and ^***^P < 0.001 for difference from each group. (*N* = 6–8).

**Figure 5:**
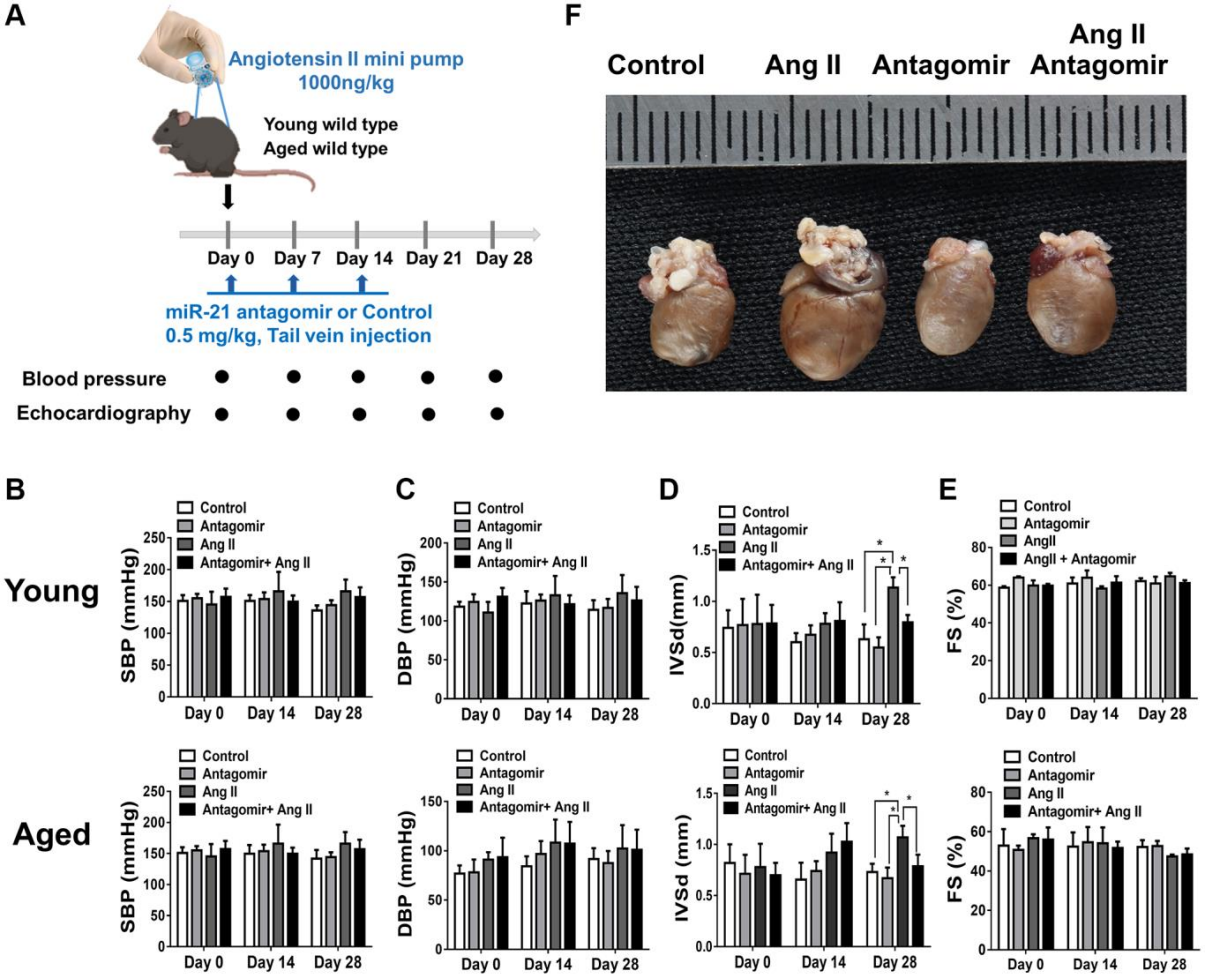
**The treatment of miR-21 antagomir mitigated angiotensin II (Ang II)-induced cardiac hypertrophy, especially in the aged mice.** (**A**) The study design investigating the effects of miR-21 antagomir in young (12 week-old) and aged mice(50 week-old) of Ang II-induced pressure overload. The sequential changes of (**B**) systolic, (**C**) diastolic blood pressures, echocardiography derived (**D**) intraventricular septal thickness at diastole (IVSd), and (**E**) fractional shortening (FS) in young and old mice treated with miR-21 antagomir or not under Ang II-induced pressure overload. (**F**) The comparison of harvested hearts in mice of control, Ang II, miR-21 antagomir and Ang II+ miR-21 antagomir. ^*^P < 0.05 for difference from each group (*N* = 4–6).

To determine whether suppression of miR-21 could prevent hypertension-induced cardiac alterations at different ages, using Ang II micropumps, we further established hypertensive models in young (12 week-old) and aged (50 week-old) miR-21^−/−^ mice compared with the control (Figure 3A). The systolic and diastolic blood pressures significantly increased in both wild-type and miR-21^−/−^ mice post Ang II treatment (Supplementary Figure 5A–5C). The body weight significantly decreased in both wild type and miR-21^−/−^ mice post Ang II treatment while there was no significant change in heart rate (Supplementary Figure 5D, 5E). Cardiac function in mice was measured by serial echocardiography. Despite no significant difference in the FS between the wild-type and miR-21^−/−^ mice in either young or aged mice (Figure 3B, 3C), compared with the wild-type, miR-21^−/−^ mice had decreased IVSd and increased LVIDd in both young and aged mice subjected to Ang II (Figure 3D, 3E). At the end of the study, the ratio of heart to body weight and the ratio of heart/tibial length indicated a decreased heart size in both young and aged miR-21^−/−^ mice post Ang II treatment compared with those of wild-type mice (Figure 4A–4C). Masson’s trichrome staining for fibrosis showed that cardiac fibrosis at day 28 post Ang II treatment was increased in both wild-type and miR-21^−/−^ mice at young and old ages compared with that in the control. Notably, Ang II-triggered cardiac fibrosis was attenuated in miR-21^−/−^ mice in both young and aged mice compared to wild-type mice (Figure 4D, 4E). Alternatively, using primarily isolated cardiomyocytes from wild-type and miR-21^−/−^ mice, we found that as miR-21 was abolished, Ang II-induced cardiac hypertrophy was attenuated (Figure 4F). For purposes of therapeutic interventions, we attempted to investigate whether suppressing miR-21 expression could mitigate pressure overload-induced cardiac hypertrophy, especially in aged subjects. Using the miR-21 antagomir, we similarly found that post Ang II induction, mice treated with the miR-21 antagomir repressed Ang II-induced cardiac hypertrophy in both young and aged mice (Figure 5). The effect was independent of the changes in blood pressure. Taken together, our findings indicated that the deletion of miR-21 improved pressure overload-induced cardiac hypertrophy in mice.

### miR-21 regulated cardiac hypertrophy associated with S100a8/NF-κB/calcineurin/NFAT pathways

**Figure 6:**
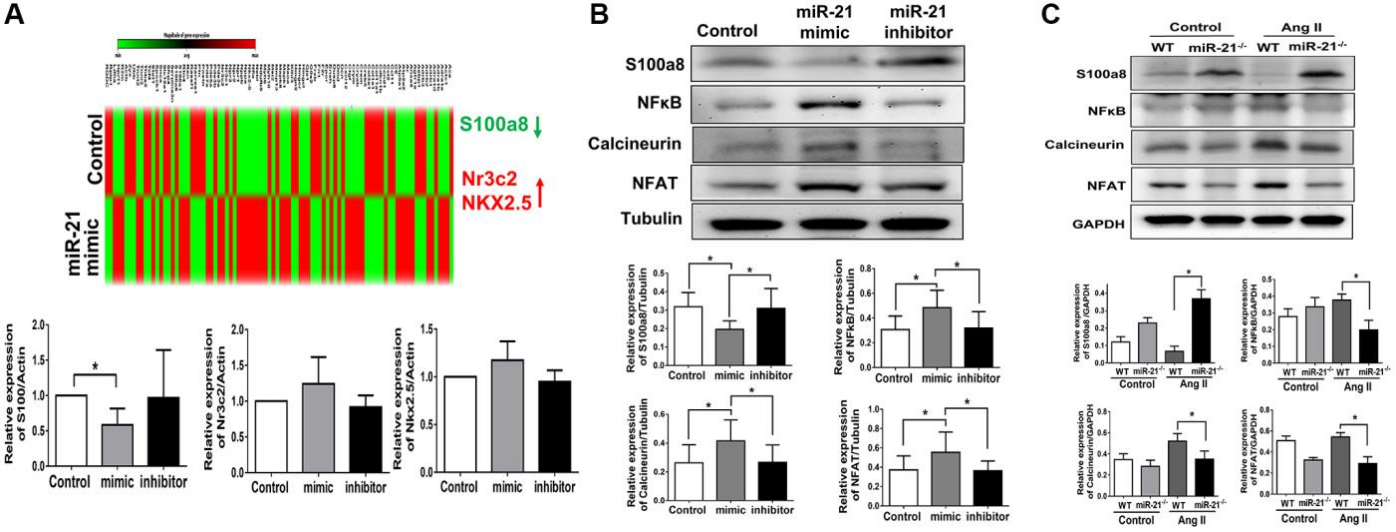
**miR-21 decreased s100a8 expression in primarily isolated cardiomyocytes.** (**A**) Heat map of cluster analysis showed dynamic changes of cardiac specific genes after miR-21 mimic treatment compared with the control. The levels of S100a8, Nr3C2 and NKX2.5 were measured by qRT-PCR in primary cardiomyocytes transfected with miR-21 mimic and inhibitor for 24 hours. (**B**) The protein expressions of S100a8, NFκB, calcineurin, and NFAT were measured by Western blot in primary cardiomyocytes. (**C**) The relative expression level of each protein was quantified by densitometry. miR-21 knockout (miR-21^−/−^) increased S100a8 and decreased NFκB, calcineurin, and NFAT expression in heart tissue of mice after angiotensin II (Ang II) treatment. The representative expressions and quantifications of S100a8, NFκB, calcineurin, and NFAT in wild type (WT) or miR-21^−/−^ of mice were detected by Western blot. ^*^P < 0.05 for difference from each group. (*N* = 4–6).

Based on our hypothesis, under aging conditions, an increase in miR-21 expression may lead to an augmented response to pressure overload, resulting in the upregulation of cardiac fibrosis and hypertrophy. A cardiac-specific qPCR array was then performed to analyze the miR-21-associated changes in gene profiles in primarily isolated cardiomyocytes. As shown in Figure 6A, three cardiac-specific genes are aberrantly regulated (fold change ≥ 2.0 and a *P* value < 0.05). Treatment with a miR-21 mimic significantly inhibited the expression of S100a8, an inflammation-associated protein that regulates the development of multiple cardiovascular diseases. Also, nuclear receptor subfamily 3 group C member 2 (Nr3C2), associated with mineralocorticoid receptor, and NK2 homeobox 5 (NKX2.5), associated with fibrosis, were upregulated as miR-21 was over-expressed. To confirm these findings, using qPCR we measured the message RNA expressions of abovementioned genes while only S100a8 was altered upon the up or down-regulation of miR-21. As determined by Western blot, S100a8 was downregulated by miR-21 mimics but upregulated by miR-21 inhibitors in primarily isolated cardiomyocytes (Figure 6B). In contrast, given that Volz et al. previously reported that S100a8 aggravates post ischemic heart failure through activation of NF-κB signaling [18], we further measured the downstream proteins associated with S100a8, including NF-κB, calcineurin, and NFAT. NF-κB, calcineurin, and NFAT were upregulated by miR-21 overexpression but downregulated by the miR-21 inhibitor in primarily isolated cardiomyocytes (Figure 6B). Taken together, the results indicated that miR-21 increased hypertrophy-associated proteins, such as calcineurin and NFAT by inhibiting the expression of the S100a8/NF-κB pathway.

Next, to further validate our findings, we investigated the cardiac expression of S100a8 and its associated proteins in miR-21^−/−^ mice. Notably, although Ang II treatment suppressed the expression of S100a8, abolishing miR-21 significantly upregulated S100a8 expression (Figure 6C). Correspondingly, cardiac hypertrophy-associated proteins, including the expression of NF-κB, calcineurin and NFAT, were significantly decreased in miR-21^−/−^ mice compared with wild-type mice in response to Ang II.

### The clinical and echocardiographic characteristics of young and aged subjects with or without hypertension

**Figure 7:**
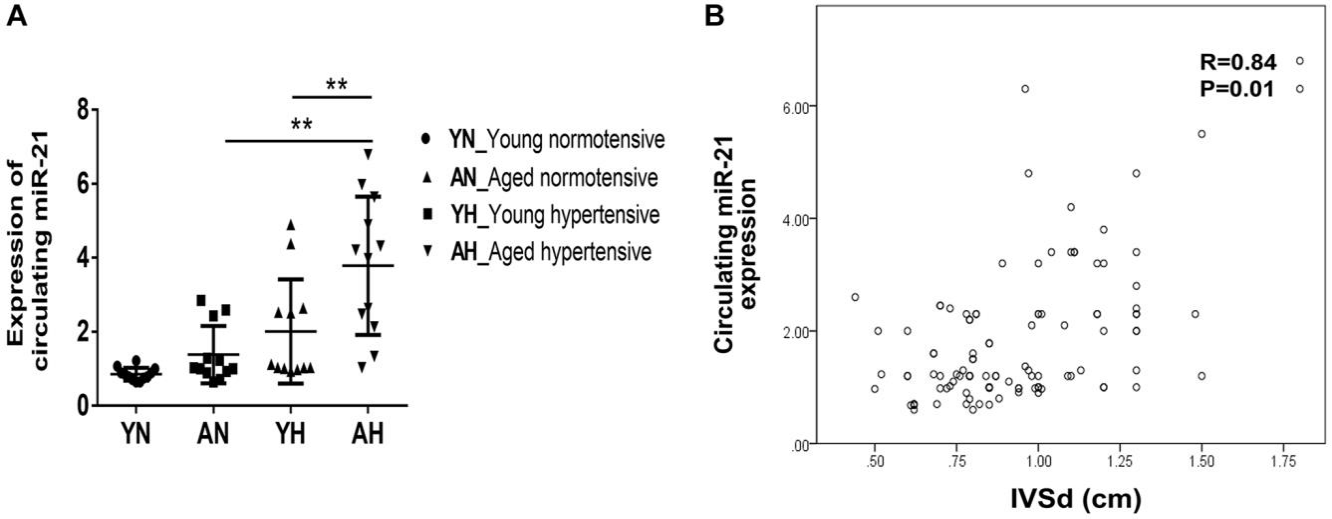
**High expression of circulating miR-21 in aged hypertensive subjects.** (**A**) Circulating miR-21 expression in normotensive young, normotensive old, hypertensive young and hypertensive old subjects. (**B**) The linear correlation between intraventricular septal thickness at diastole (IVSd) and circulating miR-21 expression in hypertensive subjects. ^**^P < 0.01 for difference from each group.

To evaluate whether the higher expression of miR-21 could also be observed in old patients with hypertension, we prospectively collected the echocardiographic information and sera of normotensive and hypertensive patients at various ages. Among 108 patients, the average ages of the young and aged subjects were 50.4 ± 10 y/o and 69.9 ± 5.9 y/o, respectively (Supplementary Table 2). For subjects with and without hypertension, the average systolic blood pressures were 153.1 ± 14.9 mmHg and 124.2 ± 8.6 mmHg, while the diastolic blood pressures were 88.5 ± 14.6 mmHg and 74.1 ± 7.2 mmHg. There were significantly increased interventricular septal thickness at diastole (IVSd) and left ventricular mass index (LVMI) in both young and aged hypertensive subjects compared with normotensive subjects. Additionally, the tissue Doppler-derived e’, a reflection of diastolic function, was more significantly decreased in hypertensive subjects, especially in aged hypertensive subjects, than in normotensive subjects. Notably, despite hypertrophic changes in both young and aged hypertensive patients, only aged hypertensive patients presented with significantly high expressions of circulating miR-21, which positively correlated with IVSd (Figure 7A, 7B).

**Figure 8:**
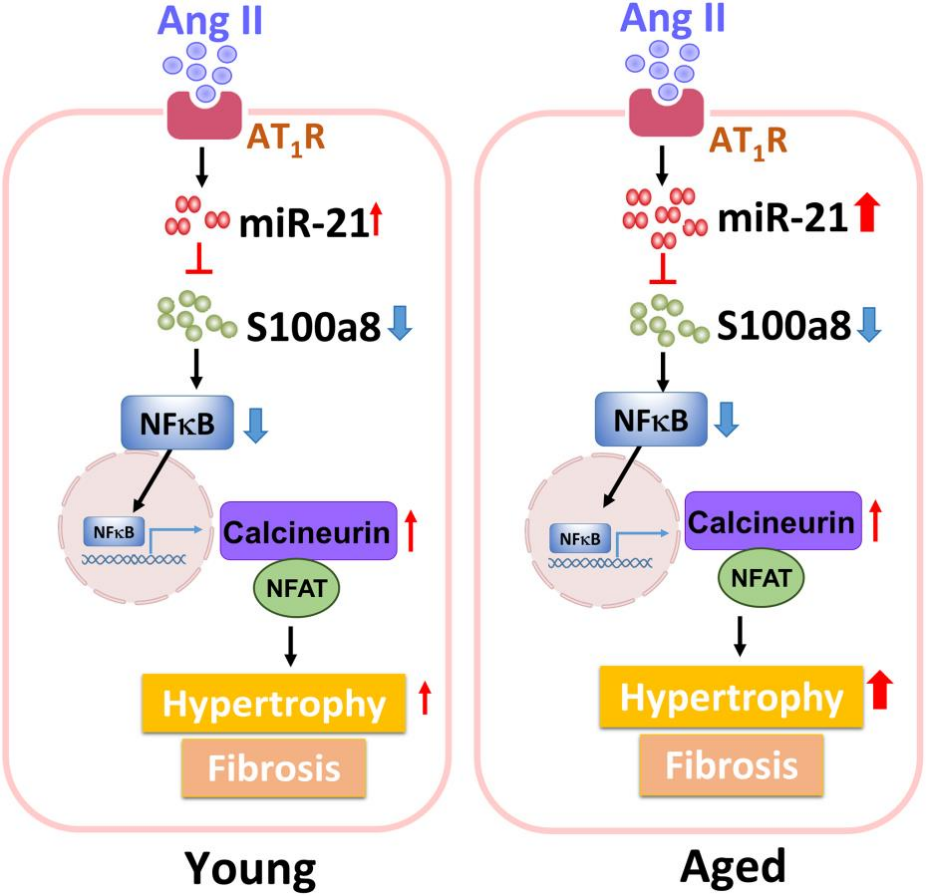
The summary of miR-21 regulation in the cardiac hypertrophy under pressure overload.

Collectively, our findings showed that, through inhibiting S100a8, miR-21 triggers cardiac hypertrophy and fibrosis under pressure overload pathophysiology. Also, circulating miR-21 could be a biomarker reflecting the hypertrophic changes in the young and aged hypertensive patients. The regulatory mechanism of miR-21 in young or aged hearts in response to pressure overload is summarized in Figure 8.

## DISCUSSION

With a high prevalence, hypertension causes systemic organ damages including myocardial hypertrophy and dysfunction [1, 4, 5]. Upon the emerging evidences including the SPRINT and the STEP trials [19, 20], the target of blood pressure is not anymore 140/90 mmHg. Instead, aggressive managements of blood pressure may yield long-term cardiovascular benefits [19, 20]. However, some patients with high blood pressures do not present clinically detectable cardiac hypertrophy [21]. Conversely, some vulnerable population, especially the aged hypertensive patients, present with maladaptive and irreversible changes in the structure and the function of the myocardium even under guideline recommended managements of blood pressure [4, 16, 17, 21]. To date, there is no effective strategy to prevent cardiac hypertrophy in aged hearts with hypertension, indicating an unmet need to understand the pathological cascades triggered by hypertension.

Cardiac hypertrophy is an adaptive remodeling of the myocardium in response to hypertension [4]. We initially observed that pressure overload could trigger enlargement of the cell size in aged cardiomyocytes; however, the underlying molecular mechanism of aging-associated cardiac hypertrophy remains unclear. Herein, we investigated the role of miR-21 in hypertension-induced cardiac hypertrophy in young and aged populations. A survey from a clinical cohort helped us clearly define the association between circulating miR-21 levels and the phenotypes of cardiac hypertrophy among aged hypertensive mice. Likewise, in aged mouse hearts, Ang II also induced pressure overload and triggered a higher intensity of hypertrophic changes, along with a higher level of cardiac expression of miR-21 as compared to that in young mouse hearts. When miR-21- was abolished in mouse hearts and primary cardiomyocytes, we further found that Ang II-induced cardiac hypertrophy could be attenuated. Mechanistically, the miR-21/S100a8/NF-κB/NFAT pathway was observed to be a regulatory mechanism of pressure overload-induced hypertrophic remodeling in the aged heart.

Cardiac hypertrophy is a major determinant of mortality and morbidity in the process of hypertension [5, 22]. Thus, regression of hypertension-induced cardiac remodeling can improve the prognosis of patients with hypertension [21]. miR-21 has been found to be associated with cardiac hypertrophy and heart failure induced by ischemic heart disease, aortic stenosis or dilated cardiomyopathy [9, 23, 24]. However, only a few reports have investigated the impact of miR-21 on the pathogenesis of hypertension-induced cardiac hypertrophy [25]. Therefore, we studied the effect of miR-21 on hypertrophy-triggered cardiac remodeling. In contrast to our findings that abolishing miR-21 attenuated Ang II-induced cardiac hypertrophy independent of changes in blood pressure, a previous report found that the levels of circulating miR-21 increased in the serum of rats with hypertension, while miR-21 attenuated cardiac hypertrophy by lowering blood pressure in rats [26]. In contrast, Watanabe et al. found that miR-21 expression levels were upregulated in the serum of mice with hypertension and in hypertrophic hearts of mice induced by Ang II [25]. Likewise, Watanabe et al. also found that overexpression of miR-21 deteriorated hypertension-induced cardiac remodeling in 8–10-week-old mice and neonatal rat cardiomyocytes [25]. However, cardiac aging was associated with a higher incidence of cardiac hypertrophy under hypertension [27, 28], which specifically delineated the role of miR-21 in aging hearts facing hypertension became crucial. Herein, we revealed for the first time that miR-21 differentially triggered cardiac hypertrophy in young and aged hearts in response to pressure overload.

As miR-21 mediates cardiac hypertrophy involving S100a8, which participates in modulating the NF-κB/NFAT pathway, we found that, by suppressing S100a8, miR-21 triggers cardiac hypertrophy under pressure overload. S100a8, a member of the S100 family, is involved in inflammatory responses and immune diseases [18]. It also functions as an important regulator of other cardiovascular disorders, including hypertension [29], viral myocarditis [30], autoimmune myocarditis [31], doxorubicin-induced cardiotoxicity [32], and cardiac hypertrophy [33]. Recently, S100a8 was reported to be one of the genes that was specifically induced during the regression of cardiac hypertrophy in a myocardial hypertrophic preconditioning model [33]. Additionally, the S100a8/S100a9 complex is a useful biomarker for elderly patients with severe heart failure. Previous studies have indicated that pretreatment with S100a8 recombinant protein attenuated norepinephrine-induced cardiac hypertrophy and subsequently reduced the expression of calcineurin and NFAT [33, 34]. In the present study, using a cardiac disease-specific qPCR array, we identified the most significant change in S100a8 expression in cardiomyocytes after miR-21 mimic/inhibitor treatment. Downregulation of miR-21 increased S100a8 expression and affected its downstream NF-κB/NFAT pathway. These results indicated that miR-21 mediated cardiac hypertrophy by targeting S100a8.

### Study limitations

The foremost limitation in our study is the difference between human and animal studies. Given the difficulty to acquire human cardiac tissues, using Ang II treatment we showed that cardiac-specific miR-21 improved myocardial function and hypertrophy in mice. Instead, miR-21 might be a sensitive diagnostic biomarker for the progression of cardiac hypertrophy in hypertensive patients. Second, alternative to Ang II treatment, another pressure-overload animal model such as aortic banding should be considered to verify our findings. Notwithstanding, this study highlight the potential of miR-21 as a new approach to protect against cardiac hypertrophy, especially in aged patients with hypertension.

## CONCLUSION

In summary, our findings revealed that increased miR-21 in aged hearts with hypertension stimuli exacerbated cardiac hypertrophy by suppressing S100a8 expression. Thus, novel miR-21/S100a8/NF-κB/NFAT pathway regulatory dysfunction might contribute to the progression of cardiac hypertrophy in aged subjects under hypertension pathophysiology.

## Supplementary Materials

Supplementary Material and Methods

Supplementary Figures

Supplementary Tables
