# Supplementary material for: miR-21 upregulation exacerbates pressure overload-induced cardiac hypertrophy in aged hearts
Source: Aging (Albany NY). 2022 Jul 28;14(14):5925–45. doi: 10.18632/aging.204194 (PMC9365557; doi:10.18632/aging.204194)
Supplement: Supplementary Material and Methods [file aging-14-204194-s001.pdf]

SUPPLEMENTARY MATERIALS AND METHODS

Human echocardiography

The chamber dimensions and intraventricular septal thickness at diastole (IVSd) were measured with the two-dimensionally guided M-mode method, and using the Simpson's method the left ventricular ejection fraction (LVEF) were measured with at the apical four chamber view. These parameters were measured sequentially for calculating left ventricular mass index (LVMI), left ventricular end-diastolic volume (EDV), end-systolic volume (ESV) and left atrial volume index (LAVi). Transmitral Doppler flow velocity were obtained from an apical four-chamber view, and peak early filling velocity (e), peak atrial velocity (a), and the E/A ratio were recorded. Early diastolic annular velocity (e') and atrial annular velocity (a') were also measured for estimating the LV end-diastolic pressure (e/e'). The average of medial and lateral e/e' were used to represent the estimated intra-ventricular pressure.

Human blood sampling and analysis

Blood was collected in EDTA tubes. After being centrifuged at the speed of 3500 rpm for 15 minutes, sera were stored at −80°C until analysis. Serum biochemical tests (including Troponin I and Brain natriuretic peptide) and total RNA extraction were performed according to the protocol and prepared for the further microRNA real time-PCR.

Administration of miR-21 antagomir

The locked-nucleicacid-modified (LNA)- miR-21 antagomir (sequence 5-caacaccagucgaugggcugu) and LNA scramble control oligonucleotides were purchased from Ambion (Thermo Fisher Scientific, Waltham, MA, USA) and were resuspended into Invivofectamine™ 3.0 Reagent (Invitrogen, Thermo Fisher Scientific, Waltham, MA, USA) according to the manufacturer's instructions. After mice received with Ang II micropum, the dose of 0.5 mg/kg LNA-miR-21 antagomir were administered intravenously via tail vein at day 0, day 7, and day 14. Afterward, the cardiac function and geometry were evaluated by echocardiography till the end of experiment.

Mouse echocardiography

Mice were anesthetized with 1.5% isoflurane and imaged in the supine position and heart rates were maintained between 400–500 bpm. Using S6 (GE, Boston, MA, USA) echo machine imaging system with a 40-MHz linear probe, a standard 2D echocardiographic study was initially performed in the parasternal long-axis view for assessment of LV dimensions and systolic function. Image depth, width and gain settings were used to optimize image quality. Frame rates were > 150 Hz. By sequential echocardiography, IVSd, left ventricular internal diameter in diastole (LVIDd), ejection fraction (EF), and fractional shortening (FS) were be measured.

Histopathological characterization

After euthanasia, the hearts of mice from each group were excised for histology and molecular analyses. The weight of heart tissue and length of tibia were measured. For histopathological examination, the heart tissue was fixed in 4% paraformaldehyde and embedded in paraffin (Alfa Aesar, Lancashire, UK). The paraffin-embedded sections of mouse hearts stained with hematoxylin and eosin were examined for the measurement of morphology and hypertrophy. Masson's trichrome stained was used for evaluation of the level of fibrosis.

β-galactosidase staining

At 14 days cultured aging cardiomyocytes were fixed in 4% paraformaldehyde (0.1 M phosphate buffer) at room temperature for 15 min. Cellular senescence was detected by *β*-galactosidase staining using a kit in accordance with the manufacturer instructions (X-gal; Sigma-Aldrich; Merck KGaA, Darmstadt, Germany). The cells were observed and photographed under a microscope (Leica Science Lab., Berlin, Germany), and all blue-stained cells (positive cells) were counted. Three vision fields were randomly selected, and 100 cells were counted to calculate the senescence rate [senescence rate (%) = positive senescent cell /100 cells × 100%].

Transfection of miR-21 mimic and inhibitor *in vitro*

After glucose starvation for 6 hr, the young and aged cardiomyocytes were transfected with miR-21 mimic (5′-uagcuuaucagacugauguuga-3′), miR-21 inhibitor (5′-ucaacaucagucugauaagcua-3′) or scrambled control (QIAGEN, Hilden, Germany) at a final concentration of 5 and 20 nM using TransIT-X2® Transfection Reagent (Mirus Bio, Madison, WI, USA), respectively. At 24 or 48 hr after transfection, the cells were harvested for further study.

Measurement of hypertrophy in primary cultured cardiomyocytes

Primary cultured cardiomyocytes were cultured on a 24-well plate and received the desired treatment. After treatment, the cells were fixed with 4% paraformaldehyde and permeabilized using 0.2% Triton

X-100. The actin filaments in cells were staining with rhodamine phalloidin (Invitrogen, Carlsbad, CA, USA). The nucleus was identified with 4–6-diamidino-2-phenylindole dihydrochloride (DAPI) (Abcam, Cambridge, MA, USA). Photographs were imaged under fluorescence microscope (Olympus BX51, Olympus Optical Co. Ltd, Tokyo, Japan). Changes in cell size were quantified using Image J software.

Quantitative PCR (qPCR) array

The primary cultured cardiomyocytes were treated with or without miR-21 mimic and then total RNAs were extracted using the miRNeasy Mini Kit (#74104, QIAGEN, Hilden, Germany). The cDNAs were synthesis using RT2 First Strand Kit (#330401, QIAGEN, Hilden, Germany). Thereafter, those cDNAs were mixed with the appropriate PCR master mix buffer (RT² SYBR Green ROX qPCR Mastermix, #330523, QIAGEN, Hilden, Germany) and analyzed on specific array plates (Cardiovascular Disease RT² Profiler™ PCR Array, #330231-PARN-174ZC-12 QIAGEN, Hilden, Germany) by performed on an Applied Biosystems (ABI) 7500 under the following conditions: 95°C for 10 min, then 40 cycles at 95°C for 15 sec and 60°C for 1 min. The intensity of each gene was then normalized to the intensity of housekeeping genes. Fold changes of gene expression and heatmap were analyzed and generated by using Gene Globe Analyze (https://geneglobe.qiagen.com/tw/analyze/).

Western blot

Equal amounts of proteins were extracted for heart tissues or primary cultured cardiomyocytes and were quantified using the BCA assay (Thermo Fisher Scientific, Waltham, MA, USA). The extracted protein was separated by sodium dodecyl sulfate-polyacrylamide (SDS) gel electrophoresis and transferred onto polyvinylidene fluoride (PVDF) microporous membranes (MERCK Millipore, Burlington, MA, USA). The membrane was blocked with 5% milk and then incubated with antibodies against S100a8 (1;1000), NFκB (1;1000), NFAT (1;1000, Abcam, Cambridge, MA, USA), calcineurin (1:1000), or GAPDH (1:5000, Sigma-Aldrich Co., St Louis, MO, USA) in TBS buffer at 4°C overnight. After incubation, the membrane was incubated with horseradish peroxidase-conjugated anti-rabbit/mouse IgG (1:5,000, MERCK Millipore, Burlington, MA, USA and Sigma-Aldrich Co., St Louis, MO, USA) for 1 hour at room temperature. Signals were detected by ECL-Western blotting system (AVEGENE CHEMX 400). The intensity of the protein band was quantified by Image J software (NIH, Bethesda, MD, USA) and the results are expressed as normalized ratio to housekeeping gene GAPDH.
