# Supplementary material for: miR-21 upregulation exacerbates pressure overload-induced cardiac hypertrophy in aged hearts
Source: Aging (Albany NY). 2022 Jul 28;14(14):5925–45. doi: 10.18632/aging.204194 (PMC9365557; doi:10.18632/aging.204194)
Supplement: Supplementary Figures [file aging-14-204194-s002.pdf]

SUPPLEMENTARY FIGURES

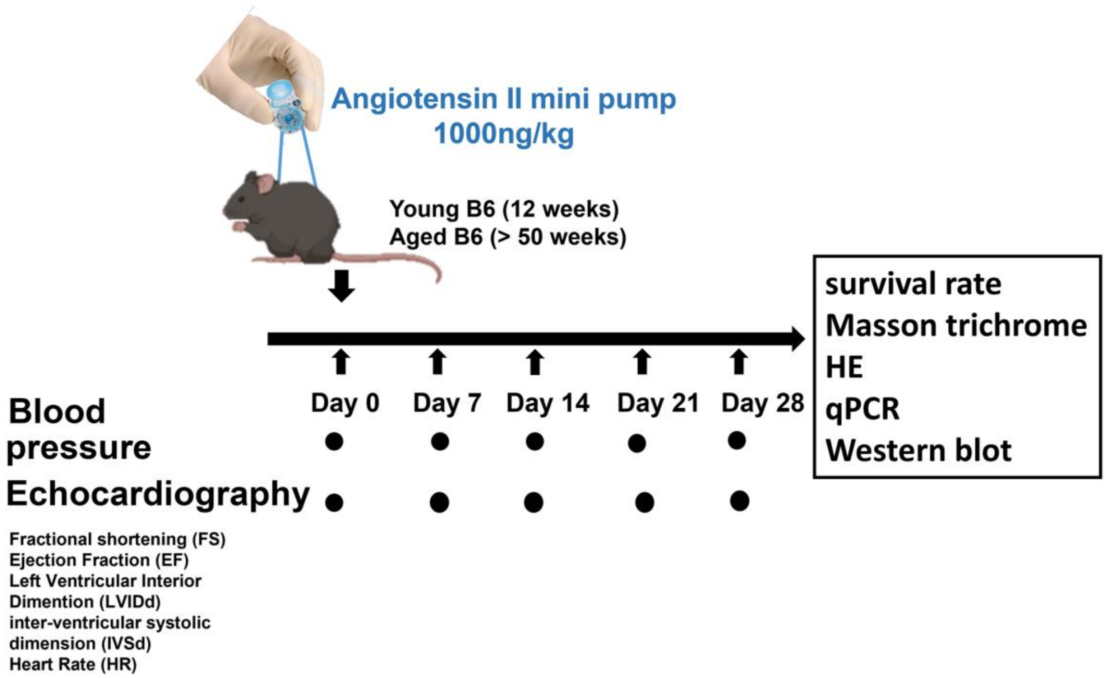

Supplementary Figure 1. The study design investigating the effects of miR-21 in young (10 week-old) and aged mice (50 week-old) of angiotensin II (Ang II)-induced pressure overload. The heart rate, blood pressure and cardiac function were measured every week. At the end of the experiments, the hearts were harvested for Masson trichrome staining for the quantification of myocardial fibrosis. The reactive molecular and protein expression were measured by qRT-PCR and Western blot, respectively.

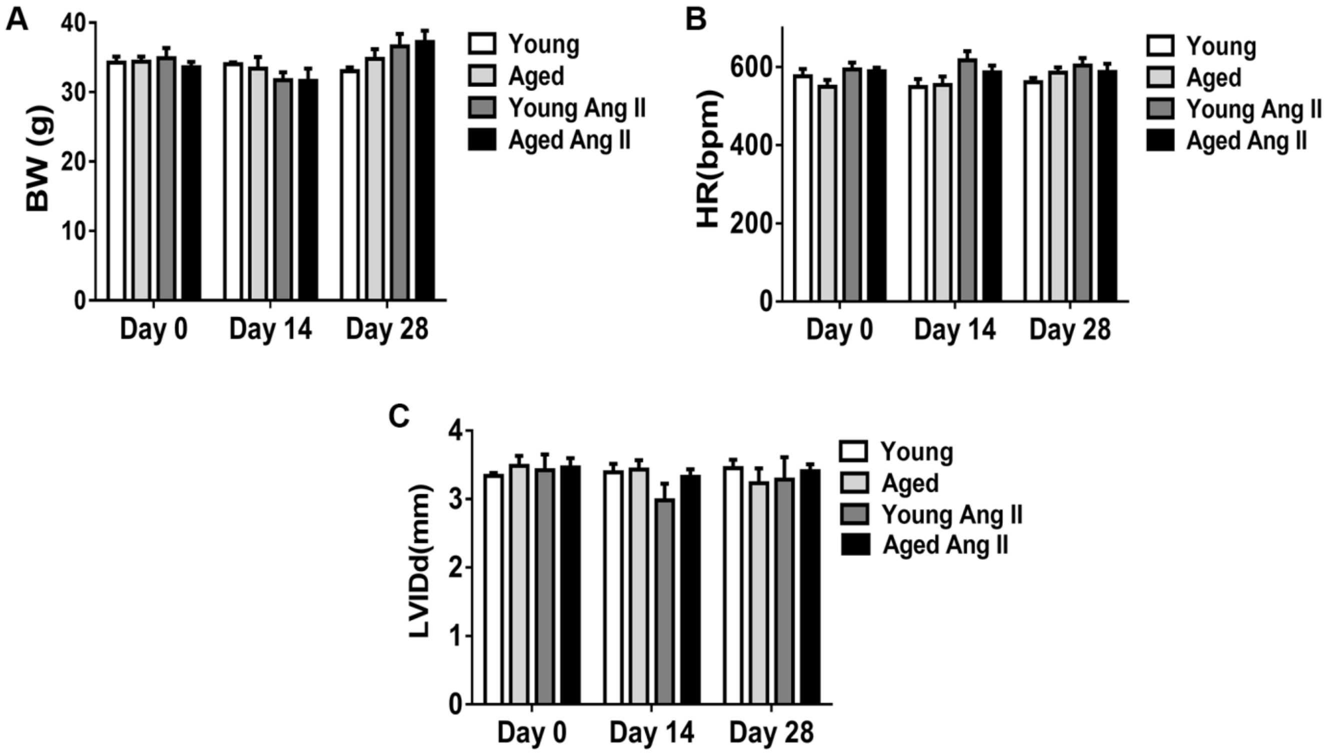

Supplementary Figure 2. Effects of angiotensin II (Ang II) on (**A**) body weight, (**B**) heart rate and (**C**) left ventricular internal diameter in diastole (LVIDd) in wild type of young and aged B6 mice. $^{*}P < 0.05$ for difference from each group. (N = 6–8).

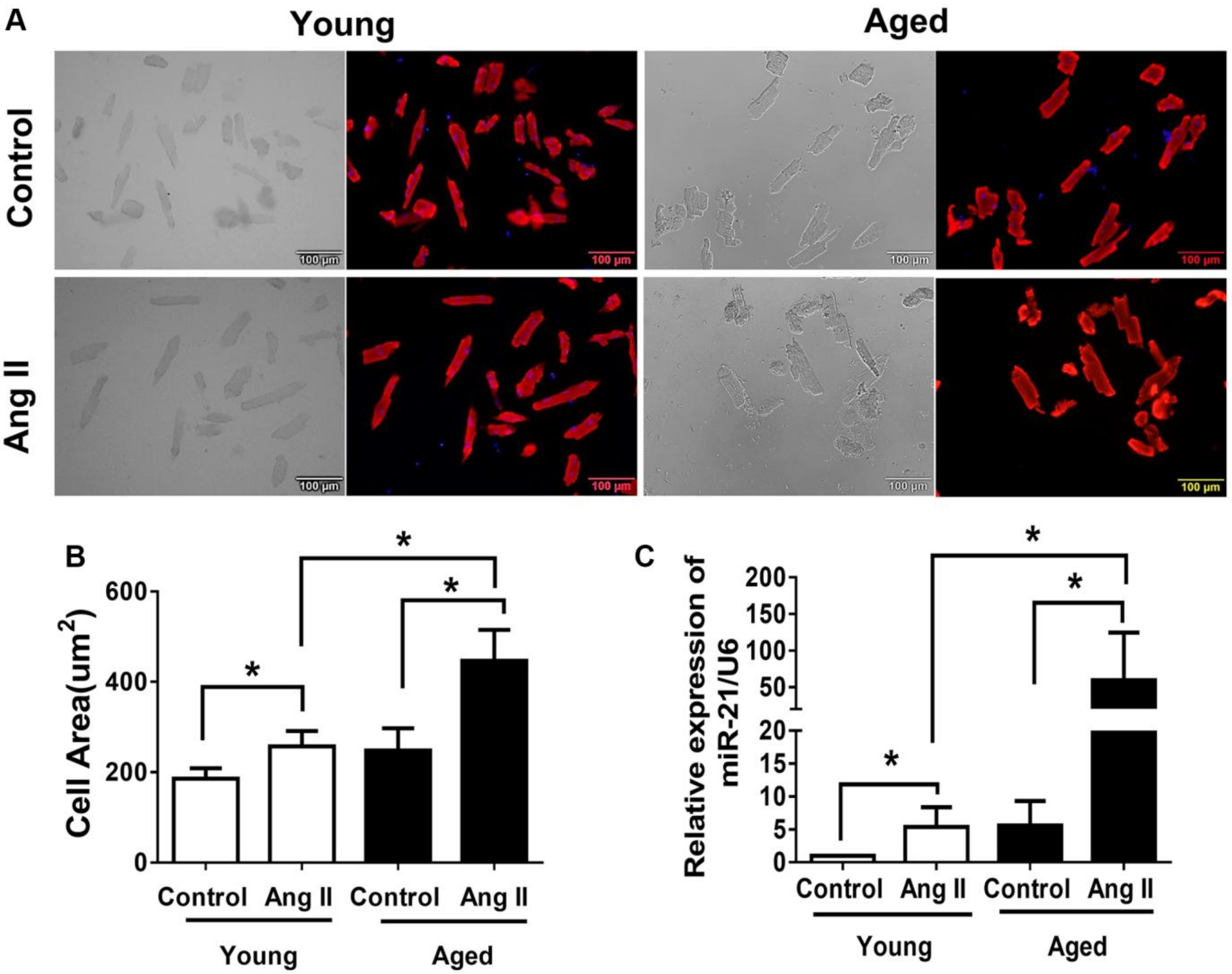

Supplementary Figure 3. Angiotensin II (Ang II) increased cardiac hypertrophy and miR-21 expression in primarily isolated mouse cardiomyocytes, especially in aged mice. (**A**) Representative merged images of F-actin immunofluorescence staining for primary cardiomyocytes isolated from young and aged mice. (**B**) Quantification of the area of primary mouse cardiomyocytes. The cell area was measured in 100 random cells in each group. (**C**) The expression of miR-21 in primary mouse cardiomyocytes was measured by qRT–PCR in each group. $^{*}P < 0.05$ for difference from each group (N = 6–8).

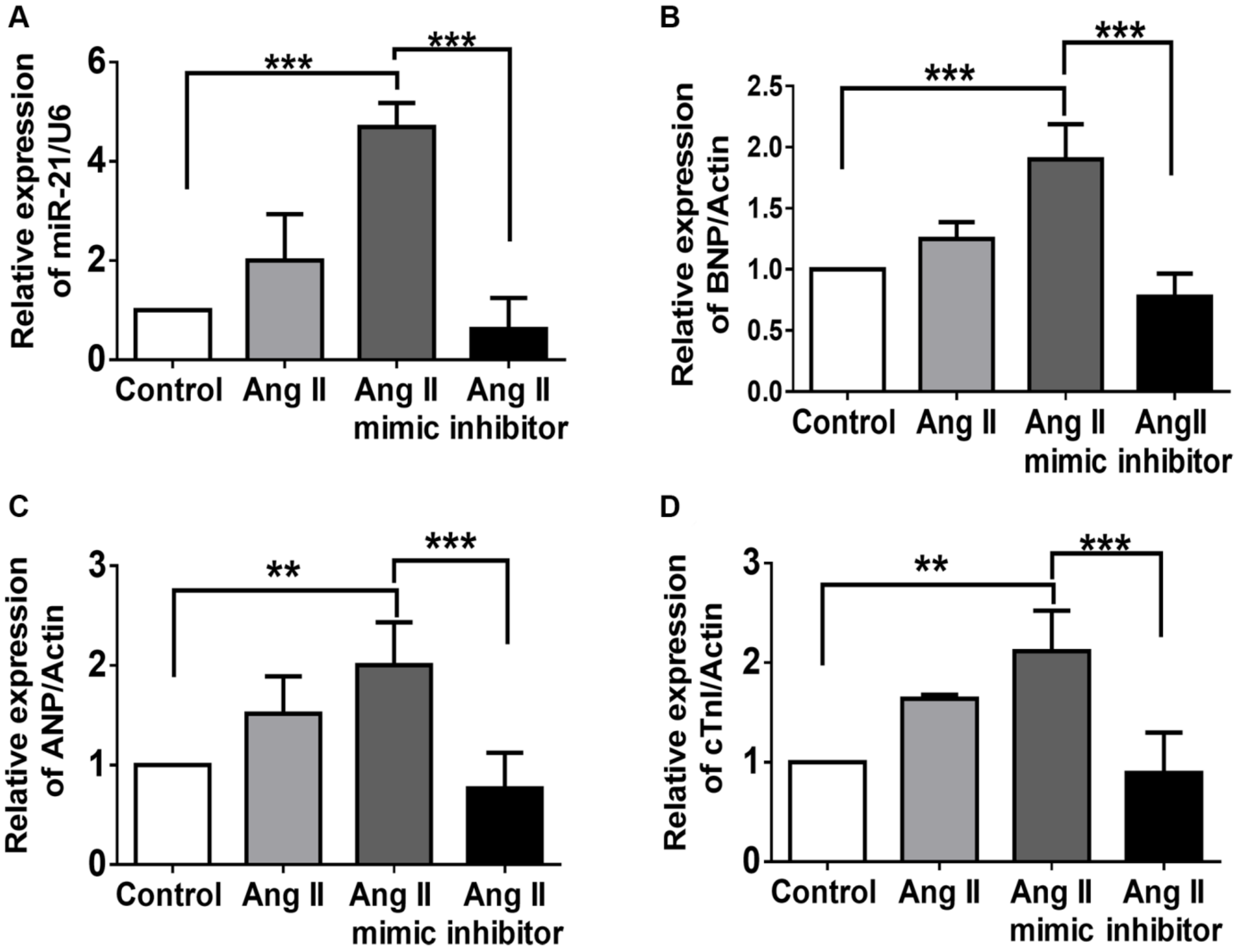

Supplementary Figure 4. miR-21 regulated the angiotensin II (Ang II)-induced cardiac injury in primarily isolated cardiomyocytes. The primarily isolated cardiomyocytes were transfected with miR-21 mimic and inhibitor for 24 hr before exposed to Ang II. The expressions of (**A**) miR-21, (**B**) BNP, (**C**) ANP, and (**D**) cTnI in primary cardiomyocytes were measured by qRT-PCR. Overexpression of miR-21 enhanced Ang II-induced cardiac injury. $^{**}P < 0.01$, and $^{***}P < 0.001$ for difference from each group (N = 4–6).

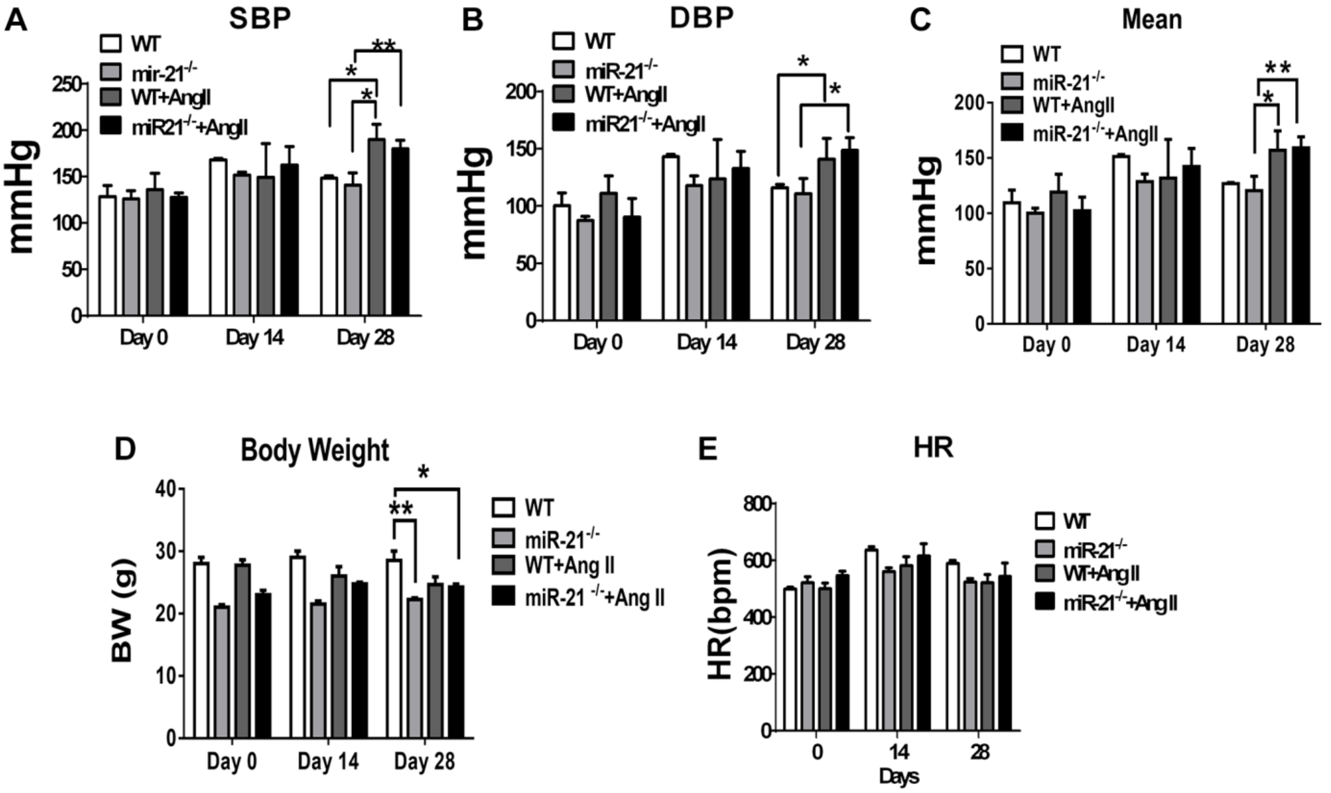

Supplementary Figure 5. Effects of angiotensin II (Ang II) on (**A**) systolic, (**B**) diastolic and (**C**) mean blood pressures, (**D**) body weights (BW), and (**E**) heart rate (HR) in wild type (WT) and miR-21 knockout (miR-$21^{-/-}$) mice. $^{*}P < 0.05$ for difference from each group; $^{**}P < 0.01$ for difference from each group (N = 6–8). Abbreviations: SBP: systolic blood pressure; DBP: diastolic blood pressure; HR: heart rate.
