# Supplementary material for: miR-21 upregulation exacerbates pressure overload-induced cardiac hypertrophy in aged hearts
Source: Aging (Albany NY). 2022 Jul 28;14(14):5925–45. doi: 10.18632/aging.204194 (PMC9365557; doi:10.18632/aging.204194)
Supplement: Supplementary Tables [file aging-14-204194-s003.pdf]

SUPPLEMENTARY TABLES

Supplementary Table 1. List of utilized primers for qRT-PCRs.

Gene	Forward primer	Reverse primer
P16^{INK4a}	accaaacgccccgaaca	gagagctgccactttgacgt
P19^{ARF}	gcagagcatgggtcgcaggttc	cgggagagggtggtggggtc
P21	cacaggagcaaagtatgccgtc	gcgaagtcaaagttccaccgt
TRF2	ggacagcgagcactaaaggc	ctggatgacaatgtctgcttc
TERT	agtggtgaacttccctgtgg	caaccgcaagactgacaaga
BNP	gtcagtcgcttgggctgt	ccagagctggggaaagaag
ANP	cacagatctgatggatttcaaga	cctcatcttctaccggcatc
MyH7	catccccaatgagacgaagt	gggaagcccttcctacagat
LDH	gcagcagggtttctatggag	tggagacagtgggattgtca
cTnl	ctctgatgctgcagattgcg	ctgccggcataggtcctgaa
S100a8	PPR 48464B-200 (QIAGEN)	
Nr3C2	PPR 44413A-200 (QIAGEN)	
NKX 2.5	PPR 43535B-200 (QIAGEN)	
GAPDH	tgattctacccacggcaatt	tgatgggtttcccattgatga
microRNA sequence		
miR-21	uagcuuaucagacugauguuga	

Supplementary Table 2. The clinical and echocardiographic characteristics of the younger (<65 years) and the older (≧ 65 years) participants (*N* = 108).

	HTN^{Neg} (*n* = 58)		HTN^{Pos} (*n* = 50)		*p*
	**Group 1: Younger (46)**	**Group 2: Older (12)**	**Group 3: Younger (40)**	**Group 4: Older (10)**	
Clnical parameters					
Age (years)	50.6 ± 9.8	69.2 ± 5.9	50.1 ± 10.3	70.7 ± 6.2	<0.001
Male	29 (63)	6 (50)	26 (65)	5 (50)	0.68
BMI (kg/m^2)	26.3 ± 2.1	25.2 ± 1.2	26.9 ± 4.1	29.6 ± 5.1	0.1
Heart rate (bpm)	69.8 ± 7.1	73.5 ± 7.3	72.6 ± 11.6	67.4 ± 8.4	0.29
SBP (mmHg)	122.9 ± 9	129.1 ± 4.8	151.5 ± 14.7	159.4 ± 14.5	0.01
DBP (mmHg)	73.3 ± 7.6	77.3 ± 4.5	88.8 ± 15.8	87.3 ± 8.9	0.02
Laboratory data					
Glucose (ac) (mg/dl)	95.33 ± 9.3	92 ± 9.4	102.1 ± 8.6	110.5 ± 19.3	0.28
eGFR (mL/min/1.73m)	93 ± 16.9	72.5 ± 13.7	80.1 ± 37.2	67.5 ± 38.2	0.29
Cholesterol (mg/dl)	178.7 ± 31.6	200.6 ± 34.5	188.7 ± 37.5	190.5 ± 42.4	0.14
Triglyceride (mg/dl)	122.6 ± 49.6	129 ± 6.1	144.6 ± 61.7	155.2 ± 65.1	0.18
Circulating miR-21 expression	1.49 ± 0.7	1.72 ± 1.1	1.82 ± 0.9	4.03 ± 2.3	0.01
Echocardiographic parameters					
IVSd (cm)	0.82 ± 0.17	0.82 ± 0.2	1.2 ± 0.39	1.42 ± 0.34	0.01

LVMI (g/m^2)	99.3 ± 30.6	92.2 ± 21.7	120 ± 65.7	138.2 ± 32.4	0.03
EDV (ml)	98.3 ± 26.6	96.33 ± 20.3	105.2 ± 37.8	112.1 ± 27.4	0.35
ESV (ml)	31.8 ± 13.1	29.8 ± 11.3	36.3 ± 16.8	34.5 ± 16.6	0.74
LAVi (ml/m^2)	24.7 ± 7.2	24.4 ± 6.2	37.1 ± 10.8	35.4 ± 3.3	0.46
LVEF (%)	76.3 ± 9.1	87 ± 1.1	66 ± 10.5	70.6 ± 9.2	0.34
E (m/s)	70.3 ± 23.6	69.9 ± 15.4	77.1 ± 16.8	71.2 ± 12.7	0.34
E/A	1.09 ± 0.43	0.89 ± 0.07	0.72 ± 0.21	0.79 ± 0.33	0.18
e' (m/s)	9.72 ± 0.85	8.15 ± 0.62	7.86 ± 0.97	5.41 ± 1.23	0.01
E/e'	7.24 ± 4.2	8.92 ± 4.2	10.11 ± 3.48	13.13 ± 2.04	0.01

Data are *n* (%) or mean ± standard error; Group differences were analyzed using analysis of variance (ANOVA). Significant differences verified using a Tukey post hoc test in single variant analysis were entered into multivariate analysis. $^aP < 0.05$, compared with Group2; $^bP < 0.05$, compared with Group3; $^cP < 0.05$, compared with Group 4; $^dP < 0.05$, compared with Group 1. Abbreviations: BMI: body mass index; SBP: systolic blood pressure; DBP: diastolic blood pressure; eGFR: estimated Glomerular filtration rate; IVSd: inter-ventricular septal diameter in diastolic phase; LVMI: left ventricular mass index; EDV: left ventricular end-diastolic volume; ESV: left ventricular end-systolic volume; E/A: trans-mitral valve E to A velocity ratio; e: early diastolic mitral inflow velocity; E/e': mitral early filling velocity to early diastolic mitral annular velocity ratio.
